# Multicall Memory in an AI Care Agent for Chronic Care Management Among Older Adults: Retrospective Observational Study

**DOI:** 10.2196/87704

**Published:** 2026-08-21

**Authors:** Markel Sanz Ausin, Akash Chaurasia, Alex Miller, Jonathan David Agnew, Rae Lasko, Mariska Raglow-Defranco, Michelle Voisard, Saad Godil, Subhabrata Mukherjee

**Affiliations:** 1 Hippocratic AI, 435 Portage Avenue, Palo Alto, CA, 94306, United States, 1 8086479626; 2 School of Population and Public Health, University of British Columbia, Vancouver, BC, Canada

**Keywords:** artificial intelligence, health care communication, patient engagement, conversational agents, memory systems, digital health, patient satisfaction, telemedicine, health informatics, machine learning

## Abstract

**Background:**

Multicall memory capabilities in AI-powered health care communication systems show promise for enhancing patient engagement, but their impact on engagement and patient satisfaction remains unclear.

**Objective:**

This study evaluated the relationship between multicall memory usage and key patient experience metrics, including call duration and satisfaction scores, in an AI-powered health care communication system.

**Methods:**

We conducted a retrospective analysis of 4415 AI care agent calls from 4189 patients using linear mixed-effects models to account for multiple calls per patient. The primary predictor was the number of memories used per call. Outcomes included call duration (in minutes), net promoter score, and patient satisfaction ratings. We analyzed the full dataset and relevant subsets (completed calls only and memory-using calls only) to assess the robustness of the findings.

**Results:**

Memory usage was significantly associated with increased call duration, with each additional memory associated with an extension of 2.47 minutes (95% CI 2.03-2.91; *P*<.001). This effect was consistent across sensitivity analyses, though it was attenuated in completed calls only (+0.54 min per memory; *P*=.004). Memory usage showed no significant association with patient satisfaction across any analysis. Given that only a small subset of calls used memories and satisfaction data were available only for completed calls, the study may have been underpowered to detect an association between memory use and net promoter score or satisfaction ratings.

**Conclusions:**

Multicall memory usage is significantly associated with enhanced behavioral engagement. The findings reveal a disconnect between engagement duration and patient-reported experience, suggesting that memory optimization strategies should focus on behavioral engagement metrics while considering factors beyond usage quantity for patient satisfaction. These results provide evidence-based guidance for health care organizations implementing memory-enabled AI communication systems.

## Introduction

### AI in Health Care Communication

The integration of AI into health care communication has rapidly evolved from experimental applications to mainstream clinical tools, fundamentally transforming how patients interact with health care systems. AI-powered conversational agents and virtual assistants now facilitate patient outreach, medication adherence monitoring, chronic disease management, and postdischarge follow-up across diverse health care settings [1,2]. These systems have demonstrated value in addressing health care accessibility challenges by providing 24/7 availability and reducing barriers to care for people in underserved populations [3,4].

Recent evidence suggests that AI-driven health care communication systems can effectively supplement traditional care delivery models. Studies have shown that these platforms are capable of conducting structured patient assessments, delivering personalized health education, and facilitating care coordination without requiring direct clinician involvement [5,6]. Zhang et al [7] demonstrated the effectiveness of virtual agents in maintaining continuity across extended health care interventions, while Dosovitsky et al [8] reported sustained user engagement with AI chatbots for individuals with depression over 14 months. The scalability of such systems offers significant potential for addressing growing health care demands while maintaining quality patient engagement. However, the effectiveness of these interactions has traditionally been limited by their episodic nature, with each patient encounter treated as an isolated event without reference to previous conversations or established rapport [9].

### The Promise of Persistent Memory in AI Systems

Longitudinal memory capabilities represent a significant advancement in AI health care communication, enabling systems to retain and reference information across multiple patient interactions. Evidence indicates that AI health assistants with longitudinal memory tend to foster greater patient trust, rapport, and engagement than systems limited to single-session interactions. Bickmore and Schulman [10] described that incorporating interaction history strengthens interpersonal bonds, while Jo et al [11] and Laban et al [12] reported that memory-enabled systems increase self-disclosure and emotional support.

Enhanced engagement appears in higher session frequencies and longer usage durations. For example, Sinha et al [13] reported an average of 33.3 sessions for more than 8 weeks, and Stein and Brooks [3] documented 103 sessions per user for more than 15 weeks, with McFadyen et al [14] observing 2.4 times more frequent and 3.8 times longer sessions compared to nonmemory counterparts. These findings suggest that persistent memory may address one of the fundamental limitations of current AI health care communication systems, namely the lack of continuity that characterizes meaningful health care relationships.

The theoretical foundation for memory-enhanced AI interactions rests on established principles of therapeutic rapport and patient-provider relationships. Jo et al [11] found that users of a chatbot with long-term memory perceived interactions as more personal and emotionally supportive compared to those without long-term memory. Similarly, Laban et al [12] observed increased self-disclosure to a social robot over time and reported significant positive effects on self-disclosure duration, length, and sentiment across sessions (*P*<.001 for all measures). However, implementation considerations are complex, as Cox et al [15] found that while verbatim references to past conversations enhanced engagement, users preferred paraphrased references for their naturalness and raised privacy concerns about verbatim recalls [15].

### Memory System Design

To address the gap between episodic AI interactions and the continuity required for meaningful health care relationships, we developed a large language model (LLM)–driven memory management framework that enables the AI care agent to autonomously create, update, and delete patient-specific memories across conversations. Memories encompass both clinical elements (eg, medical history, care preferences, and concerns) and nonclinical elements (eg, family background, hobbies, and goals) to support holistic patient understanding. The system incorporates content safety filtering to exclude controversial or privacy-sensitive information and uses Health Insurance Portability and Accountability Act (HIPAA)–compliant storage. In subsequent calls, stored memories are made available to the conversational model, which determines whether to reference them proactively or passively. Full implementation details are described in the *Methods* section.

### Study Rationale and Objectives

Taken together, the literature suggests that multicall memory should enhance AI health care communication through interconnected mechanisms: continuity fosters relational development, personalization increases perceived empathy, and accumulated trust promotes deeper self-disclosure. All these elements are expected to manifest as increased behavioral engagement. Despite the theoretical promise of multicall memory in AI health care communication, significant gaps remain in our understanding of optimal implementation and real-world effectiveness. While existing literature has demonstrated enhanced engagement metrics in controlled research settings, the relationship between memory usage quantity and patient satisfaction remains unclear [13,14]. The literature consistently highlights the growing convergence of ethical principles and regulatory mandates in governing AI-based patient memory systems, with privacy-by-design emerging as a foundational principle across multiple studies [16,17]. However, persistent memory in health care AI systems may compromise patient privacy and system security, as deep neural networks can retain personal information from even a single training instance, leading to unintended data leakage [18,19].

Furthermore, the assumption that increased memory usage improves patient experience lacks empirical validation. The complex interplay between engagement metrics, such as call duration and patient satisfaction measures, requires systematic investigation. Understanding these relationships is important for health care organizations implementing AI communication systems, given that resource allocation and system optimization depend on clearly defined performance metrics and patient outcome targets.

This study addresses these knowledge gaps by examining the relationship between multicall memory usage and key patient experience metrics in a real-world AI-powered health care communication system. Drawing on the continuity, personalization, and relational development mechanisms described in the literature, we hypothesized that greater memory usage would be associated with increased behavioral engagement, as measured by call duration. We further examined whether memory usage was associated with improved patient satisfaction, as measured by net promoter score (NPS) and patient ratings, to test whether the relational benefits of memory translate into patient-reported experience. By analyzing actual patient interactions rather than controlled research settings, this investigation provides evidence-based guidance for health care organizations implementing memory-enabled AI communication systems.

## Methods

### Study Design

We conducted a retrospective observational analysis using mixed-effects modeling to evaluate the relationship between multicall memory usage and patient engagement and satisfaction outcomes. The study used individual calls as the unit of observation, with patients having varying numbers of calls over the study period. This approach allowed us to account for the clustering of multiple calls within individual patients while examining call-level predictors and outcomes.

Data were collected from May 28, 2024, to March 25, 2025, during routine operations of an AI-powered health care communication system. The mixed-effects framework was selected to address the hierarchical structure of the data, where calls were nested within patients, and to control for patient-level characteristics that might influence both memory usage patterns and outcome measures.

### Ethical Considerations

This study used fully anonymized and deidentified datasets derived from routine operational data collected by the AI care agent system. All personally identifiable information was removed prior to analysis. According to the US Department of Health and Human Services, under the Common Rule (45 CFR 46.102[l]), research involving only deidentified data does not constitute human participants research, as there is no intervention or interaction with living individuals and no access to identifiable private information [20]. Specifically, the data met the “safe harbor” standard of deidentification outlined in the HIPAA Privacy Rule § 164.514(b)(2), with all 18 types of identifying information removed or excluded [21]. No formal application for institutional review board assessment was submitted, as the analysis of fully deidentified, nonhuman participant data did not meet the regulatory threshold requiring institutional review board review under these federal policies. Strict data security protocols were followed, and all relevant institutional policies regarding responsible data management and research integrity were adhered to throughout the study. No individual patient consent was required.

### Patient Population

We included adult patients (aged ≥18 y) who had at least one interaction with the AI care agent system for chronic care management (CCM) during the study period. Patients were excluded if they had incomplete demographic data.

The study population encompassed patients from 4 US geographic regions (West, Northeast, Midwest, and South) who engaged with the AI system through both inbound and outbound calls. These patients primarily had chronic conditions requiring ongoing management, including diabetes, hypertension, and heart disease.

We collected demographic data on age, sex, and regional distribution. Participation in memory-enabled interactions was determined by system defaults and individual consent preferences.

### Intervention

The AI care agent system was designed to conduct comprehensive patient interactions over the phone, including inbound (where the patient calls the AI health assistant) and outbound (where the AI health assistant calls the patient) CCM onboarding and routine care management check-ins. The system used natural language processing and conversational AI to engage patients in structured yet personalized dialogues.

Multicall memory functionality enabled the AI agent to retain and reference information from previous conversations with each patient. “Memories” were defined as discrete pieces of information retained from previous calls, such as patient-reported symptoms, medication concerns, lifestyle factors, care preferences, or nonclinical information. Each memory represented a specific data point that the AI could reference in subsequent interactions. The system used guardrails to ensure protections and patient consent.

Memory usage was quantified as the number of memories actively referenced during each call, ranging from 0 (no memory usage) to 22 memories per interaction. The system’s memory use algorithm prioritized conversationally and clinically relevant information, with specific optimizations to improve the smoothness of memories; to avoid awkward, forced, or irrelevant memory usage; and to provide clinical utility (such as recalling a patient’s clinical preferences or environmental factors that could affect their care).

Calls were classified as “completed” if the patient remained on the line throughout the full AI interaction protocol and “partially completed” if the patient disconnected before the intended conclusion of the call.

### Memory System Architecture

The multicall memory system was implemented as a modular component within the Polaris 3.0 (Hippocratic AI, Inc) AI care agent platform. The system used fine-tuned versions of the *LLaMA 3-405B Instruct* (Meta Platforms, Inc) model for both the conversational interactions and the memory management operations.

#### Memory Definition and Scope

A memory is defined as a modular, self-contained piece of patient information, encompassing both clinical elements (eg, medical history, motivations, challenges, medical preferences, fears, and concerns) and nonclinical elements (eg, family background, hobbies, activities, career, aspirations, and anecdotes). For example, if a patient inquired about tracking their weight, the system could store this as a discrete memory that the AI agent could reference in subsequent conversations to recommend daily weight monitoring for fluid retention.

#### Memory Lifecycle: Chained Prompt Workflow

Memory creation, updating, and deletion are managed through an agentic, multistep workflow executed after each patient conversation using a single LLM with different prompts. The memory lifecycle process follows a multiagent pipeline where memories are updated, deleted, generated, and filtered for safety. All the prompts for these agents follow a similar prompt template, consisting of 4 sections: (1) general instruction for the task the model needs to perform; (2) few-shot examples on how to complete the task and how to format the output; (3) the conversation the AI just had with the patient, which is used as a reference for the LLM to update the memories; and (4) the previous list of memories, which is empty when it is the first conversation with the patient. The memory agents perform the following tasks in their corresponding stages:

- Review and update: The system receives the completed conversation transcript along with any existing patient memories as context. The LLM identifies existing memories requiring updates based on new information disclosed during the conversation. Updated memories are overwritten, combining prior and new information into a new memory.
- Deletion assessment: Following the update step, a second agent evaluates the remaining memories for obsolescence or contradiction with newly disclosed information. Memories containing outdated or directly contradictory content are flagged for deletion by the LLM and subsequently deleted from the system.
- New memory generation: A third agent identifies strictly new information from the conversation not captured by existing memories and proposes new discrete memories for storage. These memories are stored alongside the existing and updated memories for subsequent calls with the patient.
- Content safety filtering: Each proposed or updated memory is inspected through an additional validation step for controversial or privacy-sensitive content, including political ideologies, racial or gender-related opinions, discriminatory views, social security numbers, passwords, and security question answers. Memories containing such content are discarded.

#### Memory Storage and Retrieval

Validated memories were versioned, linked to patient records, and stored in a dedicated HIPAA-compliant database. During subsequent calls, all existing memories for a given patient were provided to the conversational model as context. The conversational model determined whether and when to reference specific memories, either proactively (eg, initiating a topic based on a stored memory) or reactively (eg, acknowledging a topic the patient raised that aligned with a stored memory).

#### Memory Usage Optimization

The system incorporated specific LLM training and prompting optimizations to improve the naturalness of memory references within conversations, including guardrails to avoid awkward, forced, or irrelevant memory usage and to prioritize clinically relevant recall.

### Outcome Measures

We assessed both engagement-related and satisfaction-related outcomes to comprehensively evaluate the impact of multicall memory usage on the patient experience. Our primary outcomes focused on behavioral indicators of patient engagement during AI interactions. Call duration was measured in minutes from call initiation to call termination, representing the total time patients spent interacting with the AI care agent. This metric captured patient engagement and willingness to participate in extended conversations with the system.

Secondary outcomes examined patient-reported satisfaction and likelihood of recommendation. NPS was collected through postcall surveys administered at the end of each completed call, asking patients, “On a scale of 1‐10, how likely are you to recommend this AI care service to another patient <INSERT PRACTICE NAME>?” NPS scores were calculated using standard methodology, with responses categorized as detractors (1-6), passives (7-8), or promoters (9-10). Patient satisfaction ratings were measured through postcall surveys using a 10-point Likert scale asking patients, “On a scale of 1 to 10, how would you rate this call?” with responses ranging from 1 (very dissatisfied) to 10 (very satisfied). These ratings provided direct feedback on the patient experience and perceived value of the AI interactions. Satisfaction measures were collected by the AI agent at the end of the call. Because NPS and satisfaction were collected only at the end of completed calls, analyses of patient-reported outcomes used a reduced sample relative to all calls.

### Statistical Analysis

We used linear mixed-effects models for 3 outcomes: call duration, NPS, and patient satisfaction. All models included random intercepts for patients to account for the clustering of multiple calls within individuals and control for unmeasured patient-level characteristics.

The primary predictor variable was the number of memories used per call. Control variables included patient age (continuous), sex (male or female), geographic region (West, Northeast, Midwest, or South), and call direction type, where inbound calls were initiated by the patient and outbound calls by the AI agent.

To assess the robustness of our findings, we conducted sensitivity analyses across samples: (1) all calls in the dataset, (2) completed calls only, and (3) calls where memories were actively used (more than 0 memories). This approach allowed us to examine whether memory effects were consistent across different analytical samples and to isolate the impact of memory usage from general call completion patterns.

Two features of the data constrained power for patient-reported outcomes: (1) memory use was rare (≈5.7%, 250/4415 of all calls; 94.3%, 4165/4415 had 0 memories), limiting exposure variation; and (2) NPS and satisfaction scores were available only for completed calls, further reducing analyzable observations (eg, 1888 for NPS; 1919 for ratings). Accordingly, null associations for satisfaction should be interpreted with caution.

Model fit was evaluated using standard criteria, including marginal and conditional *R*^2^ values for linear models. Intraclass correlation coefficients were calculated to quantify the proportion of variance attributable to patient-level clustering. Analyses were performed using SPSS version 28.

## Results

### Baseline Characteristics

The study analyzed 4415 calls from 4189 patients during the study period. Patient demographics showed a mean age of 72.1 (SD 10.2) years, with 57.2% (2398/4189) female participants. Geographic distribution included 32.1% (1346/4189) from the West region, 23.6% (988/4189) from the Northeast, 4.8% (200/4189) from the Midwest, and 39.3% (1645/4189) from the South region (Table 1).

**Table 1.** Characteristics of patients and calls.

Characteristics	Values
Total patients, N	4189
Age in years, mean (SD)	72.1 (10.2)
Sex, n (%)	
Male	1791 (42.8)
Female	2398 (57.2)
Region^a^, n (%)	
West	1346 (32.1)
Northeast	988 (23.6)
Midwest	200 (4.8)
South	1645 (39.3)
Calls	
Total calls, N	4415
Calls per patient, mean (SD; range)	1.05 (0.24; 1‐4)
Call duration, min, mean (SD)	15.5 (12.4)
Call completion rate, n (%)	2162 (49)
Call direction, n (%)	
Agent initiates	2993 (67.8)
Patient initiates	1422 (32.2)
Memory usage	
Calls with memories used, n (%)	250 (5.7)
Memories per call, mean (SD)^b^	2.6 (2.3)
Memory usage distribution, n (%)	
0 memories	4165 (94.3)
1 memory	96 (2.2)
2‐3 memories	100 (2.3)
4‐5 memories	33 (0.7)
6 or more memories	21 (0.5)
Satisfaction measures, mean (SD)	
NPS^c^^,^^d^	8.47 (2.9)
Patient rating^d^	8.79 (1.7)

a A total of 10 observations had no region associated with them.

b Among calls with memory usage greater than 0.

c NPS: Net promoter score.

d Among completed calls with available data.

Call patterns revealed substantial variation in patient engagement levels. The mean number of calls per patient was 1.05 (SD 0.24), with a range of 1 to 4 calls per patient. The mean call duration was 15.5 (SD 12.4) minutes, and the overall call completion rate was 49% (2162/4415). Mean memory usage was 2.6 (SD 2.3) memories per call among calls that used memory features. Importantly, first calls with any patient cannot use memories since none have been generated yet; only second and subsequent calls have the potential to reference previously stored memories from earlier interactions. The distribution of memory usage showed that 94.3% (4165/4415) of calls used no memories, 2.2% (96/4415) used 1 memory, 2.3% (100/4415) used 2 to 3 memories, 0.7% (33/4415) used 4 to 5 memories, and 0.5% (21/4415) used 6 or more memories. Satisfaction measures were collected only on completed calls, contributing to smaller analytic N for NPS and ratings.

Among completed calls with available satisfaction data, the mean NPS was 8.47 (SD 2.9) and the mean patient rating was 8.79 (SD 1.7). These baseline characteristics demonstrate substantial heterogeneity in both patient demographics and engagement patterns, supporting the use of mixed-effects modeling to account for individual patient variation.

### Outcomes

#### Call Duration Analysis

Mixed-effects modeling revealed a significant positive association between memory usage and call duration across all calls in the dataset. Each additional memory used during a call was associated with an increase in duration of 2.47 (95% CI 2.03-2.91) minutes (*P*<.001), representing the primary finding of enhanced engagement through memory use (Table 2).

**Table 2.** Linear mixed effects models—memory effects on call duration and patient satisfaction^a^.

Predictor	Call duration (min)	Patient satisfaction	
		NPS	Patient rating
Intercept, *β* (95% CI)	1.50 (−32.86 to 35.85)	9.15 (1.43 to 16.88)^b^	8.19 (3.53 to 12.86)^b^
Memories used, *β* (95% CI)	2.47 (2.03 to 2.91)^c^	0.062 (−0.061 to 0.184)	0.036 (−0.037 to 0.110)
Age, *β* (95% CI)	0.131 (0.095 to 0.166)^c^	−0.010 (−0.024 to 0.003)	0.004 (−0.004 to 0.012)
Sex (ref:^d^ Female)			
Male, *β* (95% CI)	1.73 (1.01 to 2.44)^c^	0.22 (−0.044 to 0.484)	0.34 (0.184 to 0.501)^c^
Region (ref: South), *β* (95% CI)			
West	1.48 (0.65 to 2.35)^c^	0.063 (−0.253 to 0.381)	−0.017 (−0.207 to 0.173)
Northeast	−1.07 (−2.01 to −0.13)^b^	−0.103 (−0.442 to 0.235)	0.176 (−0.027 to 0.378)
Midwest	−0.33 (−2.03 to 1.37)	−0.026 (−0.626 to 0.574)	0.023 (−0.342 to 0.388)
Call direction (ref: agent initiates call)			
Agent receives call, *β* (95% CI)	5.04 (4.27 to 5.82)^c^	−0.025 (−0.350 to 0.301)	−0.021 (−0.217 to 0.174)
Model statistics			
Observations, n	4415	1888	1919
Patients, n	4189	1857	1857
ICC^e^	0.334	0.334	0.334
Marginal *R*^2^	0.050	0.002	0.008
Conditional *R*^2^	0.367	0.335	0.339

a Models include random intercepts for patients to account for the clustering of calls within patients. The call duration model includes all calls. Net promoter score (NPS) and patient rating models include completed calls only.

b *P*<.05.

c *P*<.001.

d ref: reference category.

e ICC: intraclass correlation coefficient.

#### Patient Satisfaction Analysis

In contrast to the significant engagement effects observed with memory usage, patient satisfaction measures showed no significant association with memory use across any analytical approach. NPS remained flat regardless of memory quantity, with a nonsignificant coefficient of 0.062 (95% CI −0.061 to 0.184) for each additional memory used (Table 2). Similarly, patient ratings showed no meaningful relationship with memory usage (*β*=0.036, 95% CI −0.037 to 0.110). The absence of a detectable association between memory count and NPS/ratings likely reflects limited statistical power and restricted exposure variability (few memory-using calls with available satisfaction data), rather than definitive evidence of no effect, although the positive coefficient may imply a relationship not detected with our smaller sample size.

This disconnect between engagement and satisfaction measures represents a key finding, suggesting that while memory usage influences behavioral indicators of patient interaction, it does not translate to improved patient-reported experience or likelihood of recommendation.

#### Sensitivity Analyses

Robustness testing across different sample restrictions confirmed the consistency of primary findings while revealing important nuances in effect sizes (Table 3). These constraints persist across sensitivity samples; although duration models use all calls, satisfaction models necessarily rely on the reduced pool of completed calls with survey data, further limiting precision of memory-satisfaction estimates. The positive association between memory usage and call duration was strongest in the full sample (+2.47 min per memory) and among calls using memory features (+1.83 min per memory).

**Table 3.** Sensitivity analysis—memory effects across sample restrictions^a^.

Sample	Total calls	Total patients	Memory → duration, *β* (95% CI)	Memory → NPS^b^, *β* (95% CI)	Memory → patient rating, *β* (95% CI)
All calls^c^	4415	4189	+2.47 (2.03 to 2.91)^d^	+0.062 (−0.061 to 0.184)	+0.036 (−0.037 to 0.110)
Completed calls only^e^	2162	2155	+0.54 (0.17 to 0.92)^f^	+0.062 (−0.061 to 0.184)	+0.036 (−0.037 to 0.110)
Memory used >0^g^	250	242	+1.83 (3.14 to 5.73)^d^	+0.067 (−0.061 to 0.184)	−0.032 (−0.269 to 0.206)

a All models include random intercepts for patients and adjust for age, sex, region, and call direction. Satisfaction measures (NPS, patient rating) were only available for completed calls, explaining identical estimates across those samples.

b NPS: Net promoter score.

c Primary analysis including all calls with mixed effects model.

d *P*<.001.

e Analysis restricted to completed calls only.

f *P*<.05.

g Analysis restricted to calls where memories were actually used (greater than 0).

### Statistical Model Performance

Mixed effects models demonstrated an appropriate fit for the hierarchical data structure. Intraclass correlation coefficients indicated that 33.4% of the variance in outcomes was attributable to patient-level clustering. Marginal *R*^2^ values for linear models ranged from 0.002 to 0.050, while conditional *R*^2^ values ranged from 0.335 to 0.367, indicating that patient-level random effects substantially improved model fit, with most of the variance explained by between-patient differences rather than the fixed effects alone.

## Discussion

### Principal Findings

This study demonstrates a direct relationship between multicall memory usage and patient engagement in AI-powered health care communication. Our analysis of 4415 calls from 4189 patients shows that memory use is significantly associated with increased call duration, with each additional memory reference associated with an extension of the conversation by 2.47 minutes (95% CI 2.03-2.91; *P*<.001). This suggests genuine patient engagement rather than mechanical time extension, as the AI system proactively chooses when to reference memories, and patients voluntarily remain engaged in longer conversations. In contrast, memory usage showed no measurable association with patient satisfaction measures, revealing a disconnect between behavioral engagement and patient-reported experience. The robustness of the duration finding across multiple analytical approaches, combined with appropriate statistical modeling that accounts for patient-level clustering, provides health care organizations with evidence-based guidance for implementing memory features to enhance patient interaction depth.

### Comparison With Existing Literature

Our findings both align with and extend previous research on longitudinal memory in AI health assistants. The enhanced engagement metrics we observed are consistent with studies by McFadyen et al [14], who reported 2.4 times higher usage frequency with memory-enabled therapy apps, and Sinha et al [13], who documented sustained engagement over extended periods. Similarly, our call duration findings support research by Jo et al [11] and Laban et al [12], who demonstrated that memory-enabled systems increase interaction duration and emotional engagement.

However, our results diverge from previous literature regarding satisfaction outcomes. While studies such as Jo et al [11] found that users perceived memory-enabled interactions as more personal and emotionally supportive, and Stein and Brooks [3] reported high user satisfaction with AI health coaches, our analysis found no correlation between memory usage quantity and satisfaction measures. This discrepancy may reflect differences between controlled research settings and real-world implementation, or it may suggest that memory quality and relevance matter more than memory quantity for patient satisfaction.

### Clinical and Operational Implications

Our findings have several important implications for health care organizations implementing AI communication systems with memory capabilities. The consistent positive relationship between memory usage and call duration provides actionable guidance for system optimization. Health care organizations can consider memory use to support patient engagement, with each additional memory reference associated with approximately 2.5 minutes of additional conversation on average. This effect appears to reflect genuine patient investment in the interaction rather than simply longer system responses, as patients voluntarily choose to remain engaged rather than terminating calls early.

The disconnect between engagement and satisfaction suggests that memory optimization strategies should focus primarily on behavioral engagement metrics rather than assuming that increased interaction duration will automatically improve patient-reported satisfaction. This finding indicates that patient satisfaction may be influenced by factors beyond memory quantity, such as memory relevance, contextual appropriateness, or the naturalness of memory integration into conversations. Health care organizations should therefore monitor both engagement and satisfaction metrics independently when implementing memory-enabled systems, recognizing that these represent distinct aspects of patient experience that may respond differently to system modifications.

An important consideration in interpreting the association between memory usage and call duration is the possibility of reverse causality. While we posit that memory references enrich conversations and encourage patients to remain engaged longer, the alternative explanation that longer calls simply provide more conversational opportunities for the AI agent to surface stored memories is also plausible. Several features of the implementation partially mitigate this concern. First, the number of memories available during a given call is determined before the call begins, as memories are generated from prior interactions and stored between sessions; that is, they are not created and referenced within the same call. Therefore, the quantity of available memories is independent of the current duration of the call. However, the conversational model’s decision to reference available memories during a call may still be influenced by call length, as longer conversations present more natural opportunities to integrate stored information.

Second, our sensitivity analyses provide further evidence bearing on the directionality question. Among the subset of calls where memories were actively used (250/4415, 5.7%), a significant dose-response relationship persisted, with each additional memory associated with 1.83 additional minutes of call duration (95% CI 3.14-5.73; *P*<.001). If reverse causality were the primary explanation, this gradient would be expected to attenuate within a sample where all calls already involved memory deployment, as variation in call length would be the driver rather than variation in memory quantity. Additionally, when restricting analysis to completed calls only (2162/4415, 49%), where call duration is bounded by the full interaction protocol and early disconnection is removed as a confound, memory usage remained a significant predictor of duration (+0.54 min per memory, *P*=.004). While neither analysis definitively rules out bidirectionality, the persistence of a dose-response gradient within memory-using calls and the sustained association within protocol-completed calls are more consistent, with memory content actively sustaining patient engagement than with call length mechanically producing more opportunities for memory deployment.

The observational design of this study cannot fully disentangle these pathways. Future research should employ prospective experimental designs—such as randomized manipulation of memory availability (eg, selectively enabling or withholding memories across patient groups) or temporal ordering analyses examining whether early-call memory deployment predicts subsequent interaction length—to establish the directionality of this relationship.

### Limitations

Several limitations should be considered when interpreting these findings. First, the retrospective observational study design limits our ability to establish causal relationships between memory usage and outcomes. While mixed-effects modeling controls patient-level clustering and observed confounders, unmeasured variables may influence both memory usage patterns and patient outcomes.

Second, our satisfaction measures were limited to NPS and patient ratings collected only from calls where these measures were available. This restriction may introduce selection bias if there are systematic differences in which patients provide satisfaction ratings. Additionally, we lacked qualitative data on patient perceptions of memory usage, which could provide insights into the mechanisms underlying the engagement-satisfaction disconnect.

Third, the study was conducted within a single AI communication system with specific memory implementation characteristics. Our findings may not generalize to systems with different memory architectures, patient populations, or clinical contexts.

Fourth, the relatively high mean age of the study population (72.1, SD 10.2 y) warrants consideration when interpreting these findings. This age distribution is consistent with CCM program enrollment, which disproportionately serves Medicare-eligible older adults managing multiple chronic conditions. However, the predominance of older adults may influence the observed relationships between memory usage and engagement in several ways. Older patients may demonstrate different patterns of receptivity to AI-driven communication compared to younger populations, including potentially greater willingness to engage in longer telephone conversations and different baseline expectations for personalized interactions. Conversely, older adults may face greater challenges with technology acceptance and familiarity with AI systems, which could attenuate engagement effects in this population relative to what might be observed among younger cohorts. The generalizability of our findings to younger patient populations, who may interact differently with AI communication systems and for whom memory-enabled features may carry different salience, remains to be established. Future studies should evaluate memory-enhanced AI communication across broader age distributions, including younger adults with chronic conditions, to determine whether the engagement patterns observed here are age-dependent or generalizable across the lifespan.

Fifth, we could not assess the clinical relevance or accuracy of memories used during calls. The quantity of memories may be less important than their clinical appropriateness, timing, or integration quality. Future research should examine memory content and relevance in addition to usage frequency.

Finally, although our sensitivity analyses provide evidence consistent with memory usage sustaining engagement rather than call length driving memory deployment, the observational design cannot definitively establish directionality in this relationship; that is, a bidirectional dynamic that future experimental studies should address.

This study provides empirical evidence for a direct relationship between multicall memory usage and patient engagement in AI-powered health care communication systems. Our analysis demonstrates that memory use is significantly associated with increased call duration, with each additional memory reference associated with 2.47 additional minutes of patient conversation on average. This association may reflect genuine patient engagement, as patients voluntarily choose to remain in longer conversations with memory-enabled interactions, though the observational design precludes definitive causal attribution.

These findings offer practical guidance for health care organizations implementing AI communication systems. Memory use represents a concrete way for organizations to enhance patient engagement, with consistent positive effects observed across multiple analytical approaches. However, the absence of corresponding improvements in patient satisfaction measures indicates that engagement and satisfaction represent distinct aspects of patient experience that respond differently to memory implementation.

The disconnect between behavioral engagement and patient-reported satisfaction suggests that effective memory optimization requires attention to factors beyond usage quantity, such as memory relevance, contextual appropriateness, and integration quality. Health care organizations should monitor both engagement and satisfaction metrics independently when deploying memory-enabled systems.

Future research should validate these findings across different patient populations and clinical contexts while investigating the qualitative factors that influence patient satisfaction independent of memory usage patterns. As AI communication systems become increasingly prevalent in health care, understanding the relationship between memory use and patient outcomes provides an evidence-based foundation for system optimization. Our findings demonstrate that memory implementation in health care AI requires careful consideration of multiple patient experience dimensions rather than assuming uniform benefits across all outcome measures.

## Abbreviations

CCM: chronic care management
HIPAA: Health Insurance Portability and Accountability Act
LLM: large language model
NPS: net promoter score
